# Type III CRISPR-Cas complexes act as protein-assisted ribozymes during target RNA cleavage

**DOI:** 10.21203/rs.3.rs-2837968/v1

**Published:** 2023-04-27

**Authors:** Evan A. Schwartz, Jack P.K. Bravo, Mohd Ahsan, Luis A. Macias, Caitlyn L. McCafferty, Tyler L. Dangerfield, Jada N. Walker, Jennifer S. Brodbelt, Giulia Palermo, Peter C. Fineran, Robert D. Fagerlund, David W. Taylor

**Affiliations:** 1 Interdisciplinary Life Sciences Graduate Programs, University of Texas at Austin; 2 Department of Molecular Biosciences, University of Texas at Austin; 3 Department of Bioengineering and Department of Chemistry, University of California, Riverside; 4 Department of Chemistry, University of Texas at Austin; 5 Microbiology and Immunology, University of Otago, PO Box 56, Dunedin 9054, New Zealand; 6 Bioprotection Aotearoa, University of Otago, PO Box 56, Dunedin 9054, New Zealand; 7 Genetics Otago, University of Otago, PO Box 56, Dunedin 9054, New Zealand; 8 Center for Systems and Synthetic Biology, University of Texas at Austin; 9 LIVESTRONG Cancer Institutes, Dell Medical School, University of Texas at Austin

## Abstract

CRISPR-Cas systems are an adaptive immune system in bacteria and archaea that utilize CRISPR RNA-guided surveillance complexes to target complementary RNA or DNA for destruction^1–5^. Target RNA cleavage at regular intervals is characteristic of type III effector complexes; however, the mechanism has remained enigmatic^6,7^. Here, we determine the structures of the *Synechocystis* type III-Dv complex, an evolutionary intermediate in type III effectors^8,9^, in pre- and post-cleavage states, which show metal ion coordination in the active sites. Using structural, biochemical, and quantum/classical molecular dynamics simulation, we reveal the structure and dynamics of the three catalytic sites, where a 2’-OH of the ribose on the target RNA acts as a nucleophile for in line self-cleavage of the upstream scissile phosphate. Strikingly, the arrangement at the catalytic residues of most type III complexes resembles the active site of ribozymes, including the hammerhead, pistol, and Varkud satellite ribozymes. Thus, type III CRISPR-Cas complexes function as protein-assisted ribozymes, and their programmable nature has important implications for how these complexes could be repurposed for applications.

## Introduction

Many bacteria employ type III clustered regularly interspaced short palindromic repeats (CRISPR)-Cas (CRISPR-associated) systems as an adaptive immune response to infection^1–3^. Upon binding an invasive RNA transcript, type III CRISPR-Cas complexes induce cyclic oligoadenylate (cOA) production by the palm domain of Cas10^10–13^. Cyclic oligoadenylates are allosteric activators of ancilliary factors including proteases and the Csm6 and NucC nucleases, which bolster the immune response^10,11,14,15^. Furthermore, binding of a non-self RNA target initiates ssDNA cleavage using the HD domain of Cas10^16–18^. After hybridization to the crRNA, type III CRISPR-Cas complexes also cleave bound RNA transcripts with unique periodicity by multiple Cas7 subunits (every 6 nucleotides for III-A, III-B, and III-E systems)^4,6,19^. Cleavage and release of a non-self RNA target arrests cOA production and ssDNA cleavage. The RNA-dependent activation of accessory nucleases by cOAs has recently been exploited for a range of sequence-specific diagnostic tools, including SARS-CoV-2 detection^20–22^.

Type III CRISPR complexes have been proposed as ancient ancestors of CRISPR-Cas systems, with Cas10 predicted as the first CRISPR-associated protein^23^. These systems are also widespread, particularly in archaea, and diverse in terms of their gene composition and organization. This evolutionary diversity has recently been highlighted by the type III-E system, which contains an operon that features a single polypeptide effector consisting of multiple Cas7 subunit domain fusions, including one domain split by a large insertion^8,9^. However, the type III-E system lacks the Cas10 and Cas5 subunits characteristic of cOA production and crRNA binding in other type III systems, respectively. While the evolutionary pathway between the well-characterized multi-subunit type III effector complexes and the III-E effector is not entirely understood, the type III-D systems may represent an evolutionary intermediate^8,9^. The type III-D systems are marked by the presence of *csx10* (a specific variant of *cas5*) and often have a *csx19* gene of unknown function. One previous report highlighted the evolutionary progression from multi-gene systems (III-D1) to the single-subunit type III-E system^8^. Recently, a variant type III-D system (III-Dv) was annotated with multiple gene fusions, suggesting its role as an evolutionary intermediate between the multi- and single-subunit type III effectors (Extended Data Fig. 1a)^9^. Type III-Dv also includes the large insertion interrupting the terminal *cas7* gene observed in the type III-E operon^8,9,24^.

Despite a fascinating evolutionary relationship between these different type III systems, the catalytic mechanism for RNA-guided RNA cleavage by type III systems remains unknown. Here, we present cryo-electron microscopy (cryo-EM) structures of the type III-Dv complex in four distinct states: bound to a crRNA (binary, surveillance complex), bound to an RNA non-self target in a pre-cleavage state, bound to a self-target with coordinated magnesium in a pre-cleavage and post-cleavage states. Through analysis of structural rearrangements between the binary and RNA target-bound complex, we show how structural rearrangements create a strict seed requirement for RNA binding and activation. We demonstrate programmable RNA cleavage at three separate active sites across three unique Cas7 subunits. Careful examination of the active sites of our structure uncovers conserved acid-base catalysis that facilitates in line scissile phosphate self-cleavage by the 2’-OH group positioned one nucleotide downstream on the RNA target, displaying remarkable similarities to known ribozymes.

## Results

### The type III-Dv effector forms a 332 kDa complex with no repeated subunits

**Fig. 1 |:**
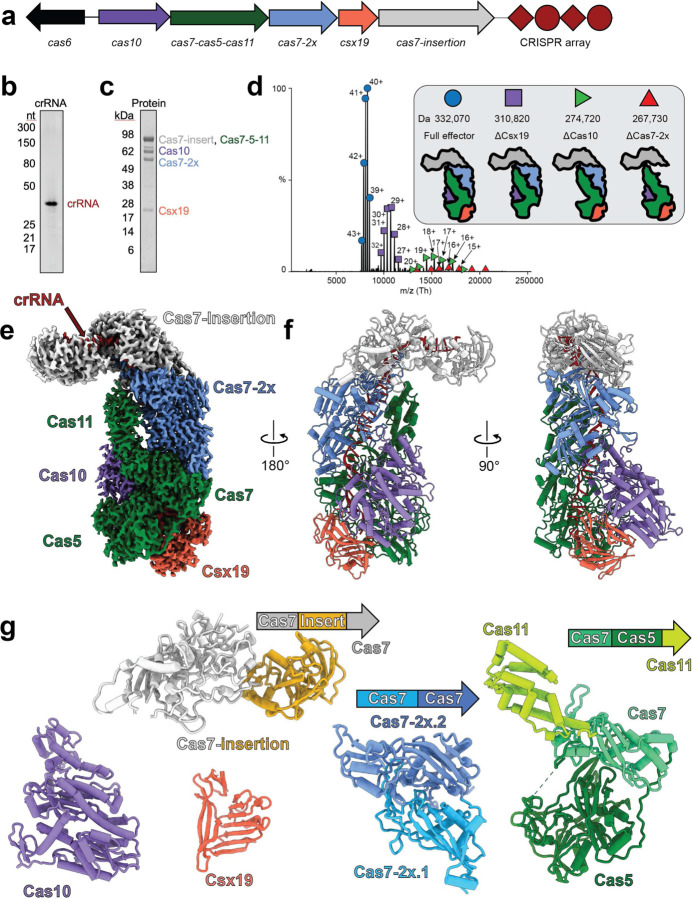
Stoichiometry and architecture of the *Synechocystis* CRISPR-Cas type III-Dv effector. **a,** Gene organization of the type III-Dv operon. Subunits and nucleic acids are colored as follows: *cas10,* purple; *cas7-cas5-cas11*, green; *cas7–2x*, blue; *cas7-insertion*, white. Cas6 is not found in the structure or purification and is thus colored black. **b,** TBE-Urea PAGE analysis of crRNA length for the type III-Dv complex. The crRNA product is 37-nt long. **c,** SDS-PAGE of the purified type III-Dv complex after size-exclusion chromatography. **d,** Native MS-MS of the type III-Dv complex. Peaks correspond to the full WT complex (circle), ΔCsx19 (square), ΔCas10 (green triangle), and ΔCas7–2x (red triangle). Ionization states are labelled for each peak. **e,** Cryo-EM map of type III-Dv binary complex. Subunits are colored as in the operon. **f,** Atomic model of the type III-Dv binary complex. **g,** Models of each subunit in the type III-D complex, colored by domains.

The operon of the type III-Dv complex from *Synechocystis* sp. PCC6803 contains *cas10*, a *cas7-cas5-cas11* fusion, a double *cas7* fusion (*cas7–2x*), *csx19*, and an insertion-containing *cas7* (*cas7-insertion*). Adjacent to the *cas* operon is *cas6–2a,* adaptation genes, and a CRISPR array containing 56 spacers ranging in size of 34 to 46 bp^25,26^. To determine the composition of the type III-Dv complex, we cloned the *cas* operon, *cas6–2a,* and first repeat-spacer-repeat of the CRISPR array from *Synechocystis* and expressed them in *E. coli* (Fig. 1). The complex was purified using metal affinity and size-exclusion chromatography, where it eluted at ~330 kDa (Extended Data Fig. 2). Analysis of the purified complex by SDS-PAGE and mass spectrometry confirmed the presence of all proteins except for Cas6–2a, consistent with other multi-subunit type III complexes^6,7^ (Fig. 1c,d Supplementary Table 1). The presence of Csx19 indicates this protein is a core component of the III-Dv complex. Urea-PAGE analysis showed a single mature crRNA with a length of 37-nt (Fig. 1b), as previously reported for type III-Dv crRNAs extracted from *Synechocystis*^25^. Native mass-spectrometry revealed that a single copy of each subunit assembled into a 332 kDa complex (Fig. 1d, Extended Data Table 1), in contrast to type III-A and -B complexes which contain multiple copies of Cas7 and Cas11 subunits^12,13,27–30^. The equal stoichiometry of subunits in the type III-Dv complex may explain why they assemble around a fixed length crRNA, while other type III complexes can assemble around crRNAs of varying lengths^6^.

### Structures of the type III-Dv surveillance complex

We determined the cryo-EM structure of the type III-Dv surveillance complex containing the 37-nt crRNA at a global resolution of 2.5 Å, enabling us to fit and refine AlphaFold2-predicted models and rapidly generate a complete atomic model for the complex (Fig. 1e-g, Extended Data Table 2 and Fig. 3,4). The overall architecture largely resembles the recently determined type III-E^31,32^ surveillance complexes, with the insertion domain protruding from the top of the complex (Extended Data Fig. 1b). A notable exception is the presence of the Cas10 and Csx19 subunits at the base of the complex. An uncharacterized domain comprising the N-terminal 112 residues of the Cas7-insertion subunit was absent from our map, likely due to flexibility. Despite the different subunit stoichiometries from other type III complexes, our structure revealed that the fused subunits still arrange into a repeating backbone around the crRNA, a structural feature conserved across all class 1 CRISPR-Cas complexes. Furthermore, all Cas7, Cas5, Cas11, and Cas10 domains within the type III-Dv complex align very well with their type III-A and III-B counterparts (Extended Data Fig. 4i-k). Running along the major Cas7 filament is the Cas11 domain of Cas7-Cas5-Cas11 and the C-terminus of Cas10, both of which are highly alpha-helical and resemble canonical small subunits^33,34^. Csx19, a subunit unique to III-D1 and III-Dv, contains multiple β-sheets and is nestled at the base of the effector between Cas5 and Cas10 subunits, making contacts with the crRNA, and contributing to the structural integrity of the complex (Extended Data Fig. 4f,g). Indeed, affinity purification of a ΔCsx19 complex with an N-terminal Cas10 tag did not result in pulldown of the complex, indicating that assembly of Csx19 onto the III-Dv complex is essential for complex assembly and stability (Extended Data Fig. 2b,d).

It has been hypothesized that the type III-Dv complex represents an evolutionary intermediate between the multi-subunit type III-D1 system and the recently described single polypeptide type III-E system (Extended Data Fig. 1)^9^. Our structure supports this notion, as the organization of the type III-D1 operon is maintained and subunits are physically fused together through flexible ~20-residue linker polypeptides. This is exemplified by the Cas7-Cas5-Cas11 subunit, which adopts a tortuous topology that places Cas7 in the body of the complex, Cas5 below it, and Cas11 back on top of Cas7 (Fig. 1g). While this demands long linkers connecting the domains, one could hypothesize that this enables the modular folding of each Cas domain to ensure accurate complex assembly. Similarly, type III-E also possesses long linkers between Cas7 and Cas11 domains. Overall, our structure suggests that the type III-Dv complex represents an intermediate, both genetically and structurally, between multi-subunit and single-subunit type III systems.

### RNA targeting by the III-Dv complex

**Fig. 2 |:**
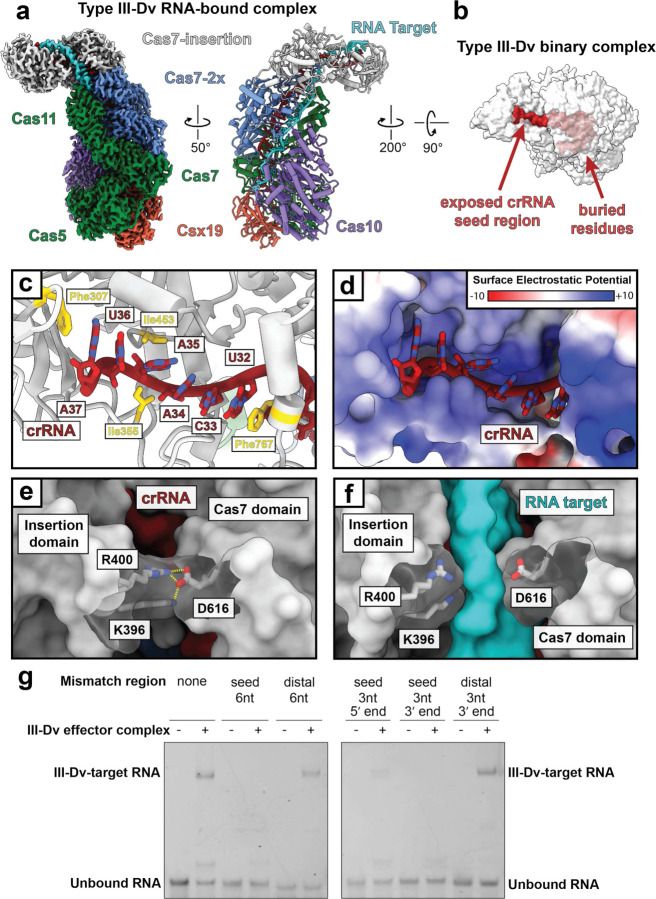
Exposed crRNA seed region initiates RNA target binding. **a,** Structure of the type III-Dv (ternary) complex bound to a target RNA, with the cryo-EM map on the left and atomic model on the right. **b,** Surface representation of the type III-Dv complex highlights the buried surface of the crRNA except for a seed region that is exposed by the insertion domain of Cas7-insertion. Units are in kT/e. **c,** Exposed residues in the crRNA seed region stabilized by residues in the insertion domain. **d,** The crRNA seed region sits in a positively charged pocket of the insertion domain. **e,** A salt bridge between D616 and R400/K396 blocks RNA target binding at this region, presumably requiring seeding first. Dashed lines show the salt bridge interactions. **f,** Separation of the salt bridge in **e** to accommodate the RNA target. **g,** Electrophoretic Mobility Shift Assay (EMSA) to test binding of RNA targets containing mismatches with the crRNA in the target 5’ seed region and 3’ end.

To understand the molecular mechanism of RNA targeting, we determined the cryo-EM structure of the III-Dv effector in complex with a 60 nucleotide (nt) target RNA at 2.8 Å resolution (Fig. 2a). The structure appears nearly identical to the binary complex, except for the presence of 34-nt of the RNA target hybridized to the crRNA. As in other class 1 complexes, every 6^th^ nucleotide of the crRNA and RNA target is flipped out by the β-hairpin thumb domain of each Cas7 domain, with the exception for the Cas7-insertion subunit^27,30,31,33,35–40^. Instead, this subunit threads an unstructured loop, 4-nt upstream of the previous Cas7, between bases U29 and C30 of the crRNA and G27 and A28 of the RNA target, pushing the stacking bases apart (Extended Data Fig. 4l). The crRNA and RNA target at this position does not have the same kinked geometry as the other Cas7 sites in the complex, suggesting that this position of the RNA target may not be designated for cleavage.

In our structure, while the target RNA engages in Watson-Crick base pairing along almost the entirety of the crRNA, after position C8 in the crRNA, the target RNA disengages at the anti-tag sequence and is funneled into an exit channel on the surface of Cas10 (Extended Data fig. 5a). This is reminiscent of non-self RNA target recognition that occurs within the Cas10 subunits of type III-A and III-B effector complexes^12,13,27^. Comparison of the target-bound complex with the binary, surveillance complex shows only minor conformational changes throughout the Cas7 backbone. However, there are notable rearrangements in the Cas10 subunit. Closer inspection of the two structures reveals an alpha helix (L238-F245) must be displaced to accommodate the 3’ end of the target RNA strand through the exit channel within Cas10, a potential feature of Cas10 activation (Extended Data Fig. 5a,b).

While most of the crRNA spacer is completely buried within the III-Dv complex, six bases at the 3’ end are highly exposed in the insertion domain of the Cas7-insertion subunit (Fig. 2b). The positive surface of the Insertion domain forms a pocket where the crRNA backbone is cradled, while the crRNA bases U(32) and A(37) are held in place through stacking interactions with F767 and F307 of Cas7-insertion, respectively (Fig. 2b-d, Extended Data Fig. 4d,e). The crRNA 3’ structure is further stabilized through hydrophobic contacts with I355 and I453. These interactions capture the 3’ end of the crRNA in a rigid conformation where the Watson-Crick faces are solvent accessible, and thus amenable to efficient base pairing. This mechanism likely shares similarities to the seeding mechanism of the type III-B complex, where the 3’ end of the crRNA must hybridize with the target RNA before the rest of the crRNA is accessible for hybridization^21^. An electrostatic network between R400, K396, and D616 within the Cas7-insertion subunit joins the cleft between the insertion domain and the Cas7 domain, limiting RNA hybridization with the crRNA (Fig. 2e). This salt-bridge is ruptured upon faithful RNA target annealing (Fig. 2f).

To test whether the exposed 3’ region of the crRNA acts as a seed for RNA targeting, we determined RNA binding to RNA targets containing various mismatches in this region through electrophoretic mobility shift assays (EMSAs). The presence of 3- and 6-nt mismatches between the RNA target and the 3’ end of the crRNA within the solvent-exposed region of the insertion domain largely prevented binding, with the seed region closest to the cleft being the most sensitive to mismatches (Fig. 2g). Conversely, mismatches at the distal, 3’ end of the RNA target had little effect on binding. These results implicate the solvent exposed region of the crRNA as the seed critical for propagation of the crRNA:TS duplex. While type III-A and -B effector complexes also rely on a 3’ seed for RNA targeting^21^, the type III-Dv complex seed is distinct in that the bases are explicitly held in a rigidified conformation amenable to base pairing. The gating mechanism described here ensures targeting fidelity by physically preventing propagation of crRNA – target duplex hybridization in the absence of seed pairing, reminiscent of Argonaute proteins and other CRISPR effectors^41,42^.

### Programmable target RNA cleavage by the type III-Dv complex

**Figure 3:**
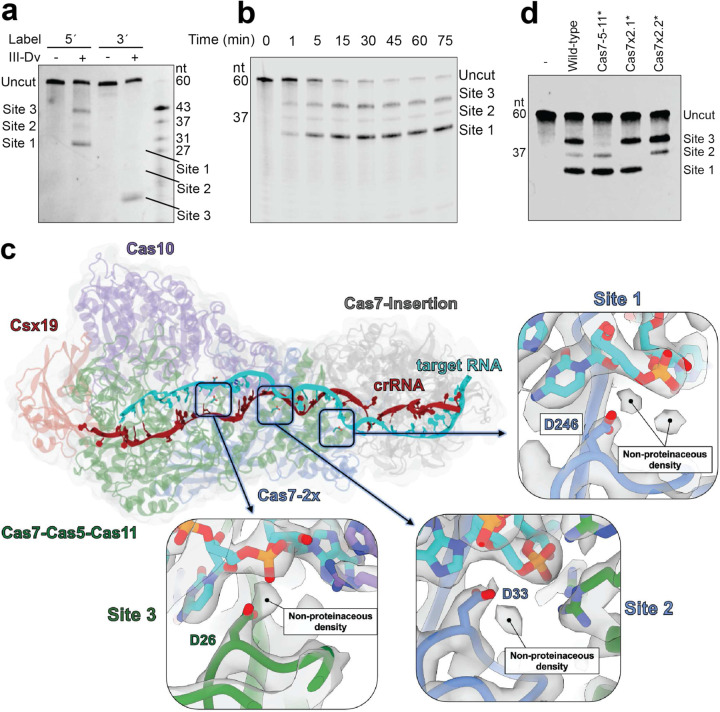
RNA targeting by type III-Dv. **a,** Cleavage of the RNA target with a 5’-FAM and 3’-FAM label. Products are visualized via TBE-Urea PAGE. **b,** RNA cleavage time course with a 5’-IRD800 labelled RNA target across 75 minutes. **c,** Overall structure of the type III-Dv complex with potential active sites mapped. Each active site contains non-proteinaceous density that may play a role in RNA catalysis. Each non-proteinaceous density is coordinated by an aspartate residue. **d,** 3’FAM-labelled RNA cleavage analysis after mutagenesis of the three active site aspartate residues of Cas7-Cas5-Cas11 (D26), Cas7–2x.1 (D33), and Cas7–2x.2 (D246).

We subsequently investigated the cleavage activity of the type III-Dv complex. Incubation of the complex with a 5’-fluorescently-labelled 60-nt RNA substrate revealed cleavage of the RNA at positions 31, 37 and 43-nt from the 5’ end (Fig. 3a, Extended Data Fig. 6a). Cleavage was rapid (Fig. 3b) and metal-dependent, with optimal cleavage occurring with Mg^2+^ and Mn^2+^ (Extended Data Fig. 6b,c). Cleavage of the same substrate with a 3’ fluorescent label revealed only one predominant cleavage event positioned 17-nt from the label (43-nt from the 5’ end), suggesting a faster rate of cleavage at this position (Fig. 3b, Extended Data Fig. 6d). Interestingly, cleavage was observed almost immediately (Fig. 3b). The observed 6-nt spacing between cleavage products and the metal dependence has previously been observed for other type III systems^6,7^. Structural analysis of other type III systems revealed a conserved Cas7 aspartate residue in proximity of the scissile phosphate that is essential for RNA hydrolysis. However, there is currently limited detail on the mechanism of RNA cleavage, including the role of divalent cations and their placement in the active site.

To investigate the mechanism of RNA hydrolysis, we froze type III-Dv complexes bound to self-target RNA onto cryo-EM grids after addition of MgCl2 and solved two structures in pre- and post-cleavage states at 3 Å and 3.44 Å resolution, respectively (Extended Data Fig. 5c). In our post-cleavage structure, the complex retains the 5’ end of the RNA target while releasing the 3’ end. Interestingly, Cas10 appears to be in the same conformation as the target-less complex, while the top of the complex perfectly aligns with the target bound structures (Extended Data Fig. 5d). This highlights an auto-inhibition mechanism whereby the 5’ end of a cleaved RNA target remains stably associated with the crRNA while the 3’ end dissociates, which would allow Cas10 to return to an inactive conformation that ceases cOA production and prevents initiation of host dormancy or death. Within our pre-cleavage structure, the Cas7 aspartate residues essential for catalysis are positioned between the scissile phosphate of the target RNA and the β-hairpin thumb, as observed for previous structures of type III complexes effectors^13,27,31^. These residues correspond to D26 of Cas7-Cas5-Cas11 (position 43 of the target), D33 of Cas7–2x.1 (position 37 of the target), and D246 of Cas7–2x.2 (position 31 of the target) (Fig. 3c). Within each active site, we observed non-proteinaceous density that is positioned adjacent to both the identified aspartate residue and the scissile phosphate that may correspond to metal ions, though we were unable unambiguously assign these densities as metal ions based purely on the structure. Individual aspartate-to-alanine mutations of these three residues were made and tested for cleavage activity (Fig. 3d, Extended Data Fig. 6e). Mutation of the predicted active site aspartate residues in the Cas7 domains successfully disrupted each cleavage event independently of the other. This feature could allow programming at these discrete and independent cleavage sites and be exploited to utilize the III-Dv complex as a programmable sequence-specific RNase.

### Type III-Dv active site aspartates coordinate divalent cations

**Figure 4:**
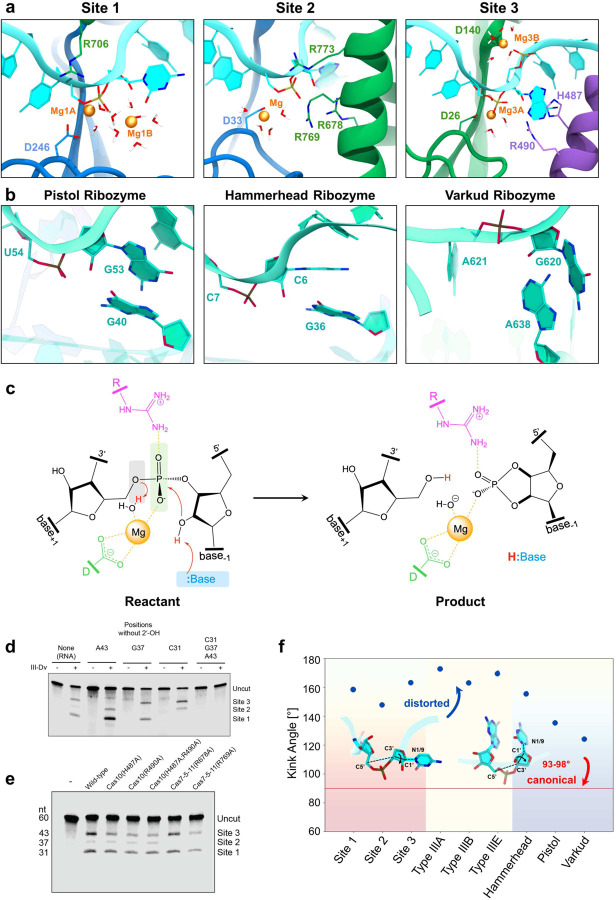
Structural basis for target RNA cleavage. **a,** Active sites (sites 1, 2, and 3) of the type III-Dv CRISPR-Cas effector complex, reporting the coordination of Mg^2+^ ions and surrounding residues, from ab-initio QM/MM MD simulations. **b,** Active site structural arrangement in the pistol, hammerhead, and varkud ribozymes, showing a flipped-out target base geometry to facilitate an in-line 2’-OH nucleophilic attack. **c,** Schematic representation of a 2’-O-transphosphorylation via acid-base catalysis proposed for target RNA cleavage. The 2’-OH group of the adjacent base acts as a nucleophile on the scissile phosphate, aided by metal ions and nearby arginine residues. **d,** 5’IRD800-labeled RNA cleavage analysis after mutagenesis of the three active sites showing deletion of nucleophilic 2’-OH at RNA positions 31, 37, and 43-nt of RNA substrate renders the substrate uncleavable at these sites. **e,** Effect of mutagenesis of residues in sites 2 and 3 on target RNA cleavage. **f,** A distortion in the target bases is reported for each site of type III-Dv complex, related type III systems (type III-A, B & E, Extended Data Fig. C2), and ribozymes. The target base flips out, positioning at a large angle from the scissile phosphate relative to A-form RNA.

We next performed classical and quantum mechanical molecular simulations to further investigate the RNA cleavage mechanism of Cas7. This approach enabled us to characterize the structure and dynamics of the active site and associated metal ions, which is unprecedented for type III CRISPR-Cas complexes. Our microsecond-long classical molecular dynamics (MD) simulations allowed Mg^2+^ ions to spontaneously diffuse and stably locate at the level of each active site (Extended Data Fig. 7). Specifically, two Mg^2+^ ions can be accommodated within sites 1 and 3 and one Mg^2+^ in site 2. Quantum mechanics/molecular mechanics (QM/MM) simulations were performed to characterize the metal ion coordination and revealed that, in all 3 sites, one Mg^2+^ ion is coordinated by an aspartate (D246 of Cas7–2x.2 in site 1; D33 of Cas7–2x.1 in site 2; D26 of Cas7–5-11 in site 3) and the scissile phosphate from the target RNA, with water molecules saturating the metal coordination sphere (Fig. 4a, Extended Data Fig 7a,b). We note that the placement of the diffused Mg^2+^ ions is consistent with the experimental cryo-EM map (Fig. 3). Indeed, the ions engage in coordinating the RNA backbone and the protein residues where weak density is experimentally observed. Abrogation of RNA cleavage through mutagenesis of these conserved aspartate residues indicates that this coordination is essential for RNA cleavage in all 3 sites of the type III-Dv complex, revealing a key, conserved role for catalytic aspartates across all type III systems (Fig. 3c, Extended Data Fig. 8).

### RNA cleavage occurs through 2’-O-transphosphorylation

The general strategies for catalyzing phosphodiester bond cleavage by nucleolytic ribozymes include the formation of an in-line positioning of the 2′-OH nucleophile (α catalysis), stabilization of the negative charge on the non-bridging phosphoryl oxygens (NPOs, ß catalysis), activation of nucleophile by a general base (i.e., γ catalysis), and facilitation of 5’ leaving group by an acidic group (i.e., δ catalysis)^43^. Accordingly, examination of each active site enabled us to speculate on the role of the chemical groups in catalysis.

In each active site, a similarly positioned Mg^2+^-bound aspartate is aided by either a positively charged Mg^2+^ ion (site 1) or arginine residue (sites 2 and 3) interacting with the NPOs of the RNA target (Fig 4a). This positioning of a 2’-OH in line with the kinked scissile phosphate is similar to a number of ribozymes, suggesting site-specific RNA cleavage by 2’-O-transphosphorylation via acid-base catalysis^43^ (Fig. 4b). In this mechanism, a general base deprotonates the 2’-OH group, activating it as a nucleophile that attacks the scissile phosphate, forming a 2’−3’-cyclic phosphate terminus and 5’ hydroxyl group as a product (Fig. 4c). Previous studies of the type III-B complex revealed the formation of 2’−3’-cyclic phosphates^4^, indicating a role of the 2’-OH in RNA cleavage. However, the results only suggested a putative mechanism. To test our proposed mechanism, we performed cleavage assays on RNA substrates with the nucleophilic 2’-OH groups removed at positions 31, 37, and 43-nt of RNA substrate. Although RNA target binding was retained, replacing the 2’-OH groups with hydrogen inhibited target RNA cleavage (Fig. 4d; Extended Data Fig. 6g), which demonstrates nucleophilic attack by the upstream 2’-OH in target RNA cleavage. This result is consistent with previous studies that showed type III complexes can bind but not cleave DNA substrates (Hale 2009, Staals 2013, Staals 2014, Tamulaitis 2014).

### Acid-base catalysis differs between the three active sites

We then scanned the active sites in our structure for catalytic acids and bases in each active site. However, despite a universal 2’-O-transphosphrylation RNA cleavage mechanism, there was no conserved base between the three RNA cleavage sites. Site 1 accommodates two Mg^2+^ ions (Mg1A, Mg1B) coordinating the scissile phosphate, but lacks protein residues and nucleobases to operate acid-base catalysis (Fig 4a). This suggests that while the first hydrated Mg^2+^ (Mg1A-OHH) could act as an acid^44^, the second (Mg1B-OH^−^) could function as a base^45^ in the catalytic process (Fig. 4c, Extended Data Fig. 7c). The coordination of Mg1B with the O5’ manifests in δ catalysis, further assisted by the Mg1B–NPO coordination that marks a ß catalysis^43^ (Extended data Fig. 7c,d). Conversely, the flipped out nitrogenous base (C31) could also serve as the catalytic base, a mechanism that has previously been observed in ribozymes^46^.

Site 2 comprises three arginine residues (R678, R769, R773 of Cas7-Cas5-Cas11) and one metal ion, which could act as an acid. R678 comes into very close contact with the nucleophilic 2’-OH and is in proximity with additional positive charges (i.e., Mg^2+^, R773, R769), which can lower its pKa and allow it to serve as a catalytic base^47,48^. Moreover, the interaction of the NPOs with R769 strengthens its role as a ß catalyst through stabilization of negative charge in the transition state and product formation. These hypotheses are supported by the loss of catalytic activity at site 2 upon R678A and R769A mutations (Fig. 4e).

Active site 3 comprises one Mg^2+^ ion and two protein residues (H487 and R490 of Cas10), with a hydrated first metal ion (Mg3A) that appears to be acting as an acid, while additional coordination with the NPO implies a second role in ß catalysis. The partial reduction in cleavage at site 3 by H487A and R490A mutant III-Dv complexes, both individually and collectively, suggests that these residues do not act as a base but may have some 2°/3° γ catalytic role^46^ (Fig. 4e). The positioning of the Mg^2+^ ions combined with mutagenesis data in site 3 suggest that the nitrogenous base (A43) may deprotonate the 2’-OH in this site, a mechanism previously described for ribozymes^46^. Interestingly, D140 in site 3 coordinates a second Mg^2+^ ion (Mg3B) on top of the catalytic center, further stabilizing the RNA backbone and serving as a ß catalyst (Extended Data Fig 7a).

### Type III RNase active sites resemble ribozymes

A comparison of the type III-Dv active site structure with the other type III complexes reveals remarkable similarities (Extended Data Fig. 8). As previously stated, all type III active sites contain a similarly positioned catalytic aspartate that is vital for RNA cleavage^12,13,27,30,31^. The arrangement of residues within the Arg-assisted active site 2 is similar to the active sites in types III-A, III-B, and site 1 of III-E complexes, and our mechanistic findings in III-Dv site 2 support previous hypotheses for the mechanism of RNA cleavage by type III-A complexes^29^. Cleavage site 3 of type III-Dv and site 2 of type III-E both contain a histidine in close proximity of the 2’-OH^13,27,31^. Beyond CRISPR-Cas complexes, the geometry of the target RNA at the catalytic sites also resembles the active sites of the hammerhead (PDB: 2OEU), pistol (PDB: 5K7C), and varkud satellite ribozyme (PDB: 5V3I) (Fig. 4a,b). This geometry favors in line nucleophilic attack of the scissile phosphate by the 2’-OH (Fig. 4f). Overall, our structural, biochemical and QM/MM data shed light on the target RNA cleavage by a 2’-O-transphosphorylation mechanism via acid-base catalysis, a mechanism that is likely conserved across all type III complexes.

## Discussion

Recent metagenomic and biochemical analysis of the type III-E single protein effector has led to hypotheses about how these systems evolved from the multi-subunit type III effectors^8,9,24^. Our structures reveal how the type III-Dv system utilizes subunit fusions while maintaining the domain organization from type III-D1^9,37^. Many of the subunits individually align well to the type III-A and type III-B subunits, but the type III-Dv complex does not vary in crRNA length or complex size. The Cas7-insertion subunit pulls the 3’ end of the crRNA away from the complex, exposing a 6-nt seed which initiates crRNA-target hybridization. We reveal the presence of the unique and uncharacterized subunit Csx19, which nestles between Cas10 and Cas5 at the base of the complex. Additionally, the presence of subunit fusions and the Cas7-insertion subunit sandwiches the type III-Dv complex between multi- and single-subunit type III complexes in the evolutionary progression of type III CRISPR systems^8,9^.

Type III CRISPR systems target single-stranded RNA, which makes them powerful post-transcriptional silencers of phage RNA^4,49^. Previous studies illustrated a 6-nt ssRNA cleavage periodicity by the III-A, III-B, and III-E effectors^6,8,19,24^. Here, we show the *in vitro* activity of the III-Dv effector complex on a ssRNA target and reveal three active sites in the Cas7-Cas5-Cas11 and Cas7–2x fusion subunits. The type III-Dv cleaves on the scale of minutes and each site can be independently controlled for cleavage, emphasizing the potential use of the III-Dv complex as a programmable RNA endonuclease.

Importantly, we report a comprehensive analysis on the acid-base chemistry of RNA cleavage by the type III-Dv CRISPR-Cas complex. The positioning of the 2’OH juxtaposed next to the scissile phosphate in all three active sites of this complex mirrors active sites of other type III complexes and shares similarities to certain ribozymes. Our data thus uncovers a 14-year mystery around the divalent cation coordination and acid-base catalysis of 2’-O-transphosphrylation by type III complexes. The type III-Dv pre-cleavage complex structure also illustrates how the notable catalytic aspartate residues in type III complexes coordinate metal ions essential for catalysis either through an active role as an acid or by stabilizing non-bridging phosphoryl groups. We identified the role of R678 in Cas7–5-11 as a catalytic base in site 2, a mechanism likely conserved across type III-A and III-B systems. The flipped-out target base is indeed a consistent feature of the type III CRISPR-Cas complexes, positioned at a ~160 º angle from the scissile phosphate, creating a geometry favorable for target RNA cleavage. While every class 1 CRISPR effector visualized to date features the flipping of a target base at every 6^th^ position^27,30,31,33,35–40^, only type III effector complexes perform RNA target cleavage^50^. Since a type III-like ancestor was the likely progenitor of other class 1 CRISPR effectors^51^, the flipped target base may be a vestigial evolutionary remnant that has persisted even when no longer required for nucleophilic attack.

## Methods

### Plasmids and oligonucleotides

Refer to Supplementary Tables 1 and 2 for lists of all plasmids and oligonucleotides used in this study.

### Culture conditions

Refer to Supplementary Table 3 for a list of all strains used in this study.

Unless otherwise noted, *Escherichia coli* strains were grown at 37°C in Lysogeny Broth (LB), or on LB-agar (LBA) plates with 1.5% (w/v) agar. Media were supplemented with antibiotics when required as follows: chloramphenicol (Cm; 25 µg/mL), and kanamycin (Km; 50 µg/mL).

### Construction of plasmids

A plasmid (pPF2434) for expression of Cas10, Cas7-5-11, Cas7-2x, Csx19 and Cas7-insert was constructed by PCR-amplifying their genes (primers PF4851+ PF4852) using *Synechocystis* genomic DNA as template and cloning the product into pRSF-1b via KpnI and PstI restriction sites. The *cas10* gene was cloned to incorporate an N-terminal His6 tag followed by TEV protease recognition sequence.

A plasmid (pPF2441) for expression of the first spacer (5’-TGTAGTAGAACCAATCGGGGTCGTCAATAACTCCCG-3’) and flanking repeat sequences (5’-GTTCAACACCCTCTTTTCCCCGTCAGGGGACTGAAAC-3’) from the type III-Dv associated CRISPR array was constructed by PCR-amplifying this region from *Synechocystis* genomic DNA (primers PF4847+ PF4848) and cloning the product into pACYCDuet-1 via NdeI and KpnI restriction sites. A plasmid (pPF2442) was constructed for expression of Cas6-2a with the first spacer and flanking repeat sequences by PCR-amplifying *cas6-2a* (primers PF4849+ PF4850) using *Synechocystis* genomic DNA as template and cloning the product into pPF2441 via NcoI and BamHI restriction sites.

Plasmids pPF3085, pPF3086, pPF3089, pPF3205, PF3436, pPF3519, pPF3521, pPF3522 and pPF3636 are for expression of mutants Cas7-2x(D29A, D31A, D33A), Cas7-2x(D241A, D246A), ∆Csx19 (nonsense mutation), Cas7-5-11(D26A), Cas10(D487A), Cas10(R490A), Cas10(D487A; R490A), Cas7-5-11(D678A) and Cas7-5-11(D769A), respectively. Plasmids pPF3085, pPF3086, pPF3089, pPF3205, pPF3436, pPF3519, pPF3521, and pPF3522 were constructed by site-directed mutagenesis through amplifying plasmid pPF2434 with primers PF5991+PF5992, PF5993+PF5994, PF6281+PF6282, PF6423+PF6424, PF6983+PF6984, PF6994+PF6995, PF6998+PF6999 and PF7000+PF7001, respectively. Plasmid pPF3636 was constructed by site-directed mutagenesis through amplifying plasmid pPF3436 with primers PF7305+PF7306. Each reaction was treated with DpnI to remove PCR template, and Gibson assembly was used to ligate the PCR product into the mutated plasmid.

### Expression and Purification of type III-Dv effector complex

Type III-Dv complex with N-terminal His6-TEV-Cas10 was expressed in LOBSTR cells containing plasmids pPF2434 and pPF2442. Five hundred mL cultures were induced with 0.5 mM IPTG at OD600 = 0.6 and grown overnight at 18°C. Cells were harvested at 10,000 x g for 10 min. The cell pellet was resuspended in 20 mL of lysis buffer (50 mM HEPES-NaOH, pH 7.5, 300 mM KCl, 5% Glycerol, 1 mM DTT, 10 mM imidazole) supplemented with 0.02 mg/mL DNaseI and cOmplete EDTA free protease inhibitor (Roche). Cells were lysed by a French pressure cell press (American Industry Company) at 10,000 psi, and the lysate was clarified by centrifugation at 15,000 x g for 15 min. The lysate was applied to a HisTrap affinity column (GE Healthcare) equilibrated in lysate buffer and eluted using a gradient against lysate buffer containing 500 mM imidazole. The fractions containing the type III-Dv complex were pooled, treated with TEV protease and incubated at 4°C during overnight dialysis in SEC buffer (10 mM HEPES-NaOH, pH 7.5, 100 mM KCl, 5% Glycerol, 1 mM DTT). The sample was applied to a second HisTrap column; however, due to inefficient TEV cleavage, the complex unexpectedly bound the column and eluted with high imidazole. The complex was further purified by size exclusion chromatography (SEC) on a HiLoad 16/600 Superdex 200 column (GE Healthcare) equilibrated in SEC Buffer. Mutant type III-Dv complexes were similarly expressed and purified, except TEV protease was omitted. Purified complexes were typically concentrated to 1.5 mg/mL using a centrifugal concentrator (Amicon; 100 kDa MWCO), aliquoted, and stored at −80°C.

### Native Mass Spectrometry

5 µL aliquots of the CRISPR complex solution were buffer exchanged into 100 mM ammonium acetate using Biospin P-6 gel columns (Bio-Rad Laboratories Inc., Hercules, CA) prior to native mass spectrometry. Samples were loaded into gold/palladium-coated borosilicate static emitters and subjected to electrospray ionization using a source voltage of 1.0 – 1.3 kV and analyzed in the positive ion mode on a Thermo Scientific Q Exactive Plus UHMR Orbitrap mass spectrometer (Bremen, Germany). Subcomplexes and ejected subunits were produced and measured via quadrupole isolation of the intact complex charge envelope, followed by higher-energy collisional dissociation (HCD) using 290 eV normalized collision energy (NCE). Ion optics and trapping gas pressure were tuned for the transmission and detection of each set of analytes, including the intact complex, subcomplexes, and ejected subunit ions. Native mass spectra were collected by averaging 500 microscans at a resolution of 1,625 at m/z 200. Spectra were deconvoluted using UniDec.

Denaturing liquid chromatography mass spectrometry (LC-MS) was performed on a Dionex UltiMate 3000 nanoLC system coupled to a Thermo Orbitrap Fusion Lumos Tribrid mass spectrometer (San Jose, Ca). The trap column (3 cm) and analytical column (30 cm) were packed in-house with polymer reverse-phase (PLRP) packing material. Approximately 80 ng of the CRISPR complex were injected and subjected to reverse-phase chromatography, utilizing water with 0.1% formic acid as mobile phase A (MPA), and acetonitrile with 0.1% formic acid as mobile phase B (MPB). Forward trapping occurred for 5 minutes at 2% MPB at a flow rate of 5 µL/min at the trap column. Elution onto the analytical column (at 0.3 µL/min) occurred by increasing MPB to 10% over a 3-minute gradient followed by an increase to 35% MPB over 32 minutes. Mass spectra were collected at a resolution of 15,000 at m/z 200, using 5 microscans and an AGC target of 1E6. Spectra were manually averaged over each subunit elution period and deconvoluted with UniDec.

### RNA cleavage by the type III-Dv effector complex

RNA targets for testing cleavage were either ordered as fluorescently labelled ssRNA (IDT) with either 5’ IRD800, 5’ 6-FAM or 3’ 6-FAM label label. All targets used for cleavage are listed in Supplementary Table 2. RNA cleavage assays were conducted in 5 µL of reaction typically containing 200 nM purified type III-Dv effector complex, 100 nM RNA substrate in final buffer conditions of 6 mM HEPES-NaOH, pH 7.5, 60 mM KCl, 5 mM MgCl2, 3% glycerol, 1 mM DTT. Reactions were incubated at 37°C for 30 min or as indicated. Reactions were stopped by adding 1 µL 6 M guanidinium thiocyanate and 6 µL 2x RNA loading dye. Samples were heated for 5 min at 95°C and immediately placed on ice for 3 min. Samples were analyzed by 1× TBE, 15% acrylamide, 8M urea denaturing PAGE (Thermo Fisher). Fluorescent probes were imaged using the Odyssey Fc imaging system (LICOR).

### Electrophoretic mobility shift assays

RNA targets for EMSAs were ordered as fluorescently labelled ssRNA (IDT) with either 5’ IRD800 or 5’ 6-FAM label. All targets used are listed in Supplementary Table 2. EMSAs were conducted in 5 µL of reaction containing 100 nM purified type III-Dv effector complex, 100 nM RNA substrate in final buffer conditions of 6 mM HEPES-NaOH, pH 7.5, 60 mM KCl, 5 mM EDTA, 3% glycerol, 1 mM DTT. Reactions were incubated at 37°C for 30 min. Samples were separated on 4% polyacrylamide (19:1 acrylamide:bisacrylamide) native gel containing 0.5× TBE at 4 °C. The fluorescent probe was imaged using the Odyssey Fc imaging system.

### Cryo-EM grid preparation and data collection

Fully assembled type III-Dv binary complex was diluted to a concentration of 0.3mg/mL in SEC buffer before 2.5 μL of sample was added to a quantifoil 1.2/1.3 grid that was glow discharged for 1 minute. Sample was applied to the grid in an FEI Vitrobot MarkIV kept at 100% humidity and 4°C before blotting for 5.5 seconds with a force of 0. For the RNA target-bound complex, non-self RNA target (PF5855) was mixed with the binary complex with a 2:1 molar ratio of RNA:binary complex for 30 minutes at 30° C in SEC buffer to a final non-self-target-bound complex concentration of 0.3 mg/mL. Pre- and post-cleavage complexes were captured by mixing self-target RNA and binary complex in a 2:1 molar ratio, followed by addition of MgCl2 in SEC buffer to a final concentration of 10mM of Mg^2+^ and 0.3 mg/mL of self-target-bound complex. Grids of the non-self- and self-target-bound complexes were frozen with identical conditions to the binary complex. Grids were loaded to an FEI Titan Krios (Sauer Structural Biology Lab, University of Texas at Austin) operating at 300kV. Images were taken at a pixel size of 0.81 Å/pixel with a dose rate of 16.2 e^−^/Å^2^/s for 5 seconds for the binary and non-self-target-bound complexes and at a pixel size of 0.8332 Å/pixel with a dose rate of 20.2 e^−^/Å^2^/s for 4 seconds using a Gatan K3 direct electron detector, giving a final dosage of ~80.5 e^−^/Å^2^ for all datasets. A Gatan Bio-continuum operating at a 20 eV slit was also used for the self-target-bound dataset. Data collection was automated using SerialEM using a defocus range of −1.2 to −2.2 µm.

### Cryo-EM data processing

Movies from the Gatan K3 were motion corrected using motioncor2, and corrected micrographs were uploaded to cryoSPARC v2^52^. After CTF correction, initial templates for template-based picking were generated using a blob picker and 2D classification. Template-based particle picking resulted in ~1.85 million particles (binary complex) and ~1.92 million particles (target-bound complex) being picked.

Processing the dataset for the binary complex was started with one round of 2D classification, sorting out particles to a new subset of ~926k particles. Ab initio reconstruction and subsequent heterogeneous refinement with four classes was utilized and ~649k particles were selected from one of the classes. Particles were then split by exposure groups before performing a final non-uniform (NU) refinement^53^, yielding a final map at 2.5 Å resolution. Using a mask generated in ChimeraX^54,55^ around the Cas7-insertion portion of the map, we reconstructed a map of this region at 2.5 Å resolution using local refinement in cryoSPARC. The two maps were stitched into a composite map using the vop maximum command in ChimeraX.

For the target-bound complex, ~1.92 million particles were input into 2D classification and filtering, sorting out particles to a new subset of ~1.07 million particles. This new subset was then input into ab initio reconstruction and heterogeneous refinement on cryoSPARC v2 with four classes and filtered out ~453k particles to a new subset of ~614k particles^52^. These particles were split by exposure groups before performing NU refinement with identical settings to the final NU refinement in the binary complex dataset^53^. The full complex model was refined identical to that of the binary complex. This refinement yielded a 2.8 Å resolution structure from ~610k particles. To refine the density for the Cas7-insertion subunit, we generated a mask in ChimeraX and performed local refinement on the ~610k particle set and reconstructed a 2.7 Å resolution map. A composite map was generated in ChimeraX using the vop maximum command.

The self-target-bound complex was processed similarly to the other two datasets. After pre-processing, ~1.28 million particle picks were filtered with 2D classification to a subset of ~690k particles. We then generated 3 models using ab initio reconstruction and heterogeneous refinement using cryoSPARC v3 and filtered the subset to ~486k particles. We input this particle set into 3D classification with 10 classes to generate a final particle set of ~182k particles and reconstructed a 3.01 Å map using non-uniform refinement of the self-target- and Mg^2+^-bound complex in a pre-cleavage state. To generate the post-cleavage structure, we utilized 3D variability analysis with 10 clusters on the ~486k particle subset. We reconstructed a 3.44 Å map using non-uniform refinement with a subset of ~41k particles from one of the 3D variability clusters.

### *In silico* subunit modeling and refinement:

The subunit models were generated using Alphafold 2 using the monomer model preset and fit into the map using Namdinator and ISOLDE^56–58^. The reduced database precision was used for the multiple sequence alignment. The AF2 job run included a relaxation step, resulting in both relaxed and unrelaxed models. The model of the full complex was refined using Phenix real-space-refinement^59^ using the model from ISOLDE as a reference turning off secondary structure restraints, NCS restraints, and local grid search. Rotamer outliers were adjusted using Coot^60^.

## Supplementary Material

Supplement 1
