# Supplementary material for: Type III CRISPR-Cas complexes act as protein-assisted ribozymes during target RNA cleavage
Source: Res Sq. 2023 Apr 27:rs.3.rs-2837968. Preprint. [Version 1] doi: 10.21203/rs.3.rs-2837968/v1 (PMC10168453; doi:10.21203/rs.3.rs-2837968/v1)
Supplement: Supplement 1 [file NIHPPRS2837968V1-supplement-1.pdf]

Supplementary Files

This is a list of supplementary files associated with this preprint. Click to download.

- Schwartzetalsuppmaterialfinal.pdf
- SchwartzetalIIIDextendeddatafinal.pdf
